# The long-term impact of a chronic total occlusion in a non-infarct-related artery on acute ST-segment elevation myocardial infarction after primary coronary intervention

**DOI:** 10.1186/s12872-021-01874-1

**Published:** 2021-01-30

**Authors:** Yu Liu, Le-Feng Wang, Xin-Chun Yang, Chang-Lin Lu, Kui-Bao Li, Mu-Lei Chen, Na Li, Hong-Shi Wang, Jiu-Chang Zhong, Li Xu, Zhu-Hua Ni, Wei-Ming Li, Kun Xia, Da-Peng Zhang, Hao Sun, Zong-Sheng Guo, Yong-Hui Chi, Ji-Fang He, Zhi-Yong Zhang, Feng Jiang, Hong-Jiang Wang

**Affiliations:** Heart Center and Beijing Key Laboratory of Hypertension, Beijing Chaoyang Hospital, Capital Medical University, No 8 of Gongren Tiyuchang Nanlu, Chaoyang District, Beijing, 100021 China

**Keywords:** Acute ST segment elevation myocardial infarction, Percutaneous coronary intervention, Chronic total occlusion, Prognosis

## Abstract

**Objectives:**

To investigate the long-term outcome of patients with acute ST-segment elevation myocardial infarction (STEMI) and a chronic total occlusion (CTO) in a non-infarct-related artery (IRA) and the risk factors for mortality.

**Methods:**

The enrolled cohort comprised 323 patients with STEMI and multivessel diseases (MVD) that received a primary percutaneous coronary intervention between January 2008 and November 2013. The patients were divided into two groups: the CTO group (n = 97) and the non-CTO group (n = 236). The long-term major adverse cardiovascular and cerebrovascular events (MACCE) experienced by each group were compared.

**Results:**

The rates of all-cause mortality and MACCE were significantly higher in the CTO group than they were in the non-CTO group. Cox regression analysis showed that an age ≥ 65 years (OR = 3.94, 95% CI: 1.47–10.56, *P* = 0.01), a CTO in a non-IRA(OR = 5.09, 95% CI: 1.79 ~ 14.54, *P* < 0.01), an in-hospital Killip class ≥ 3 (OR = 4.32, 95% CI: 1.71 ~ 10.95, *P* < 0.01), and the presence of renal insufficiency (OR = 5.32, 95% CI: 1.49 ~ 19.01, *P* = 0.01), stress ulcer with gastraintestinal bleeding (SUB) (OR = 6.36, 95% CI: (1.45 ~ 28.01, *P* = 0.01) were significantly related the 10-year mortality of patients with STEMI and MVD; an in-hospital Killip class ≥ 3 (OR = 2.97,95% CI:1.46 ~ 6.03, *P* < 0.01) and the presence of renal insufficiency (OR = 5.61, 95% CI: 1.19 ~ 26.39, *P* = 0.03) were significantly related to the 10-year mortality of patients with STEMI and a CTO.

**Conclusions:**

The presence of a CTO in a non-IRA, an age ≥ 65 years, an in-hospital Killip class ≥ 3, and the presence of renal insufficiency, and SUB were independent risk predictors for the long-term mortality of patients with STEMI and MVD; an in-hospital Killip class ≥ 3 and renal insufficiency were independent risk predictors for the long-term mortality of patients with STEMI and a CTO.

## Introduction

Previous studies have shown that approximately 40%–60% of patients with acute ST-segment elevation myocardial infarction (STEMI) have multivessel diseases (MVD), and the long-term prognosis of these patients is worse than that of patients with single-vessel lesions [1–4]. Of the patients with STEMI and MVD, approximately 10%–15% have a chronic total occlusion (CTO) in a non-infarct-related artery (IRA), and these patients have an even worse prognosis [5–7]. This study examined the factors that can impact the long-term outcome of patients with STEMI and MVD by using multifactorial analysis to compare patients with and without a CTO. The aim of the study was to provide the evidence necessary to develop reasonable treatment strategies.

## Methods

### Study population and design

All of the data considered in this study come from Beijing Chaoyang Hospital, one of the foremost centers for the treatment of coronary heart disease in China. In 1992, the Heart Center of Beijing Chaoyang Hospital established China’s first primary percutaneous coronary intervention (PCI) team, which became known for its rapid response and around-the-clock availability for patients with acute myocardial infarction (AMI). The information center of the hospital provided the diagnostic information classified according to ICD-10 code. A total of 323 subjects that presented with STEMI with MVD from January 2008 to November 2013 were enrolled retrospectively. Of the enrolled cohort, 97 subjects with a CTO in a non-IRA were included in the CTO group, and 226 subjects with MVD but no CTO in a non-IRA were included in the non-CTO group. All subjects received emergent coronary angiography, angioplasty, or both; only IRAs were treated with an emergent operation. Optimal medical therapy had been used in all of the subjects, and all subjects were analyzed and followed up to determine the long-term mortality and the incidence of major adverse cardiovascular and cerebrovascular events (MACCE) after primary coronary angiography and angioplasty. This was a retrospective study, so there was no need for patients to provide informed consent.

### Primary coronary angioplasty and revascularization

An interventional cardiologist is on-duty at all times at the Heart Center of Beijing Chaoyang Hospital, and all subjects underwent primary coronary angiography to identify the culprit vessels. Percutaneous transluminal coronary angioplasty should be performed if antegrade blood flow in the IRA could not be restored to grade 3 on the Thrombolysis in Myocardial Infarction (TIMI) scale. The chief operating surgeon determined whether stent implantation was necessary, and intervention was only IRAs were treated during the primary procedure. The revascularization of non-IRAs by means of PCI or coronary artery bypass grafting (CABG) was not considered unless there was evidence of myocardial ischemia from an ST depression on the electrocardiogram, ischemic driven arrythmia, or heart failure during hospitalization.

### Medication and assessment of cardiac function during hospitalization

All patients were given aspirin (300 mg) and clopidogrel (300–600 mg) before emergent angiography and unfractionated heparin (80–120 IU/kg) during the operation. We did not adopt ticagrelor since it was not available in our hospital and clopidogrel was applied for the subjects in both groups. Glycoprotein IIb/IIIa inhibitors were not given if the patient had a history of gastrointestinal or intracranial bleeding, a coagulation disorder, or a platelet count less than 90 × 10^9^/L. All patients in the two groups had been treated with aspirin and clopidogrel for at least 12 months, and they had received long-term treatment with β-receptor blockers, angiotensin-converting enzyme inhibitors, and statins (unless contraindicated). Besides, all the patients enrolled were treated with standard treatment in the guidelines to control the possible bias caused by the treatment approach to the long-term clinical outcome.

Patients were ranked according to the Killip classification of cardiac function as follows [8]: class 1, no evidence of heart failure; class 2, signs indicating a mild to moderate degree of heart failure (e.g., an S3 gallop, rales halfway up the lung fields, or elevated jugular venous pressure); class 3, pulmonary edema; and class 4, cardiogenic shock or hypotension. For the regression analysis, Killip classes 1 and 2 corresponded to mild heart failure, and Killip classes 3 and 4 corresponded to severe heart failure.

### Definitions

MVD was defined as the presence of at least one lesion with a diameter stenosis of ≥ 70% in at least two major epicardial coronary arteries, as determined by visual assessment by at least two chief interventional doctors in the catheter laboratory. A CTO in a non-IRA was defined as a TIMI flow grade of 0 or 1 in a non-treated vessel that was unrelated to the acute infarct episode, with or without anterograde or retrograde filling through collateral vessels [9]. The differentiation between a CTO and an acute occlusion was determined according to the morphology of the occlusion (the presence of a fresh thrombus or bridge, ipsilateral or contralateral collaterals), the results of electrocardiographic recording, and the possibility of previously documented acute coronary events in the same territory.

### Endpoints

Clinical endpoints included all-cause mortality, MACCE (the composite of death, reinfarction, target vessel revascularization for ischemia, and stroke) at the 30-day and 10-year follow-ups. All primary endpoint events were adjudicated by an independent clinical events committee that was blinded to the original allocation. Follow-up information was obtained by direct telephone interviews and outpatient visits and from the database of the Beijing Chaoyang Hospital Information Center.

The residual myocardial ischemia was based on one of any items of following indications: (1) the onset of chest pain. (2) ECG showed myocardial ischemia. (3) Myocaritdial enzymes increased again. (4) Rest and exercise myocardial radionuclide imaging. (5) Exercise treadmill test showed myocardial ischemia.

### Statistical methods

Continuous parameters with a normal distribution were presented as the mean ± SD, and the Student’s t test was used to determine the significance of differences between the mean values. Qualitative parameters were analyzed using a chi-square test. Mortality curves < 140 months after STEMI were constructed using the Kaplan–Meier method. Cox regression analysis was used to analyze the long-term mortality factors for patients with STEMI and MVD and compare patients from the CTO and non-CTO groups. The level of statistical significance was *P* < 0.05 (two-tailed). SPSS 26.0 (IBM Corp., Armonk, NY, USA) software was used for the statistical analysis.

## Results

### Baseline characteristics

There were no significant differences in the CTO and non-CTO groups in terms of age, sex composition ratio, history of PCI, history of smoking, hypertension, diabetes, hyperlipidemia, stress ulcer, or liver function abnormality (*P* > 0.05). The incidences of old myocardial infarction (OMI), renal insufficiency, and in-hospital pulmonary infection were significantly higher in the CTO group than they were in the non-CTO group (*P* < 0.05). The results of coronary angiography showed that there was no significant difference in the distribution of culprit vessels in the two groups (*P* > 0.05), but the proportion of three-vessel lesions was significantly higher in the CTO group than it was in the non-CTO group (*P* < 0.05) (Tables 1, 2).

**Table 1:** Baseline characteristics between the two groups

Variable	Non-CTO (n = 226)	CTO (n = 97)	*P* value
Age,years	60.5 ± 10.9	62.8 ± 12.3	0.092
Female (n, %)	56 (24.8)	17 (17.5)	0.151
Hypertension (n, %)	127 (56.2)	50 (51.6)	0.443
Diabetes (n, %)	74 (32.7)	31 (31.9)	0.887
Smoking history (n, %)	106 (46.9)	48 (49.5)	0.674
Hyperlipidemia (n, %)	174 (76.9)	78 (80.4)	0.385
OMI (n, %)	7 (3.1)	23 (23.7)	< 0.001
History of PCI (n, %)	6 (2.6)	3 (3.1)	0.832
Renal insufficiency (n, %)	4 (1.8)	8 (8.3)	0.005
Lung infection (n, %)	10 (4.4)	25 (25.8)	< 0.001
Stress ulcer (n, %)	6 (2.7)	5 (5.2)	0.258
Abnormal liver function(n,%)(n, %)	21 (9.3)	7 (7.2)	0.544

Data are presented as mean ± SD or n (%). OMI, old myocardial infarction; PCI, percutaneous coronary intervention

**Table 2:** Angiographic features between the two groups

Variable	Non-CTO (n = 226)	CTO (n = 97)	*P* value
Three vessel disease (n, %)	143 (63.3)	85 (87.6)	< 0.001
LM (n, %)	34 (15.0)	11 (11.3)	0.377
*Culprit vessel (n, %)*			
LAD (n, %)	94 (41.6)	31 (31.9)	0.103
RCA (n, %)	98 (43.4)	47 (48.5)	0.399
LCX (n, %)	29 (12.8)	19 (19.6)	0.118
LM (n, %)	3 (1.3)	0	0.254

LM, left main trunk; LAD, left anterior descending artery; LCX, left circumflex; RCA, right coronary artery

### Heart function and operation

**Fig. 1:**
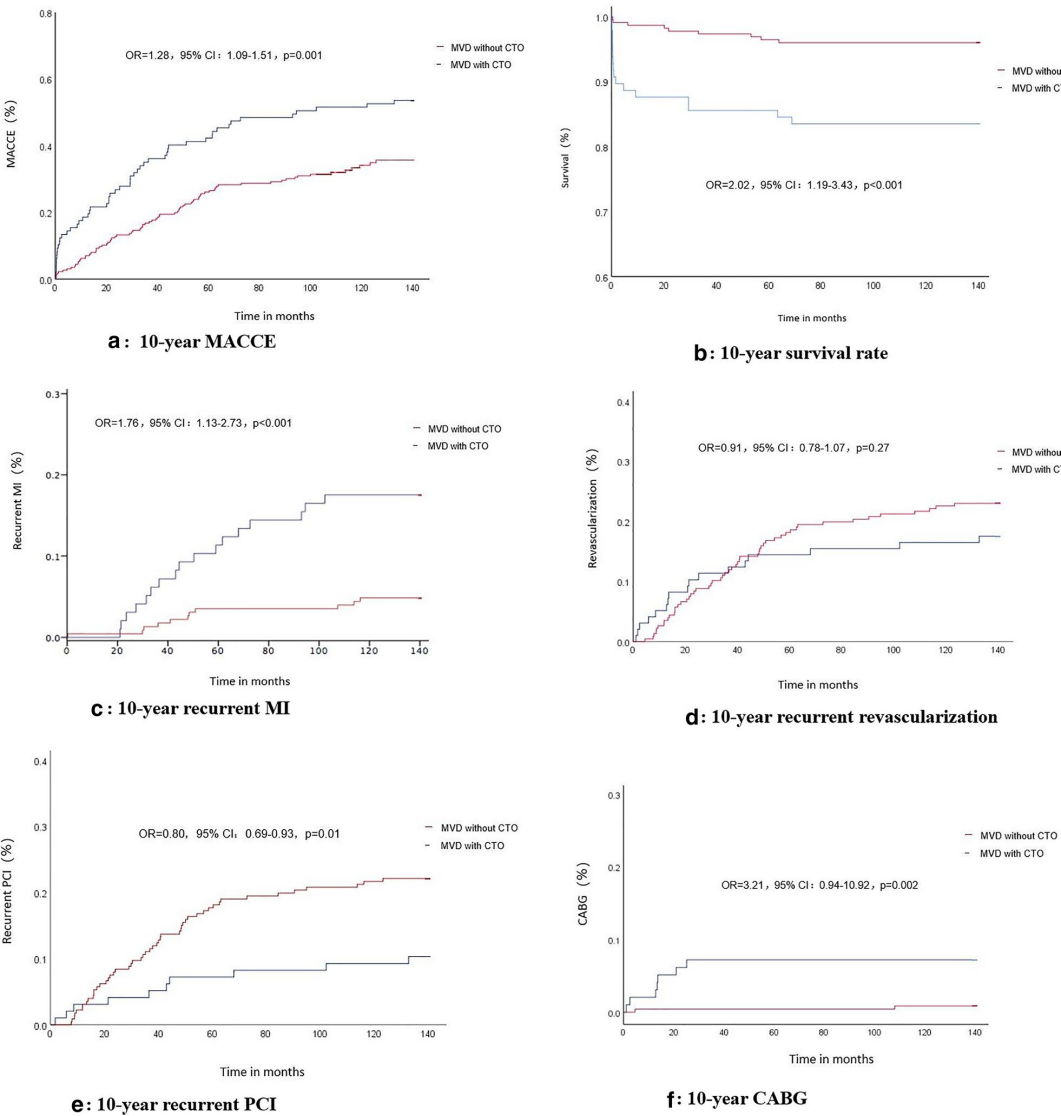
Comparison of the survival rate and the incidence of MACCE in patients with STEMI and MVD in the CTO and non-CTO groups after 10 years (**a** 10-year incidence of MACCE; **b** 10-year survival rate; **c** 10-year incidence of recurrent MI; **d** 10-year incidence of recurrent revascularization; **e** 10-year incidence of recurrent PCI; **f** 10-year incidence of CABG). MACCE, major adverse cardiovascular and cerebrovascular events; STEMI, ST-segment elevation myocardial infarction; MVD, multivessel diseases; CTO, chronic total occlusion; MI, myocardial infarction; PCI, percutaneous coronary intervention; CABG, coronary artery bypass grafting

In the present study, No-reflow occurred in 7 cases (7.2%) in CTO group and 19 cases (8.4%) in non-CTO group, who were treated by intra-coronary injection of sodium nitroprusside, verapamil respectively in the emergency PCI procedure. A TIMI blood flow grade of 3 was restored in the IRAs of all patients after the emergency PCI procedure. There was no significant difference in the number of stents implanted per patient (*P* > 0.05) in the two groups. The rate of emergency stent implantation and the proportion of Killip class ≤ 2 were significantly lower in the CTO group than they were in the non-CTO group (*P* < 0.05), while the rate of intra-aortic balloon pump implantation and the proportion of Killip class ≥ 3 were significantly higher in the CTO group than they were in the non-CTO group (*P* < 0.05).During hospitalization, the rate of CABG was significantly higher in the CTO group than it was in the non-CTO group (10.3% *vs* 1.5%, *P* < 0.001) (Tables 3, 4 and Fig. 1).

**Table 3:** Comparison of heart function and operation in the two groups

Variable	Non-CTO (n = 226)	CTO (n = 97)	*P* value
Emergency stenting (n, %)	219 (96.9)	80 (82.5)	< 0.001
Number of stent per capita	1.43 ± 0.58	1.51 ± 0.67	0.418
IABP implantation (n, %)	128 (56.6)	75 (77.3)	< 0.001
CABG (n, %)	3 (1.5)	10 (10.3)	< 0.001
LVEF(%)	50.7 ± 11.5	46.6 ± 12.8	0.101
*Killip class*			
≤ 2 (n, %)	182 (80.5)	61 (62.9)	< 0.001
≥ 3 (n, %)	44 (19.5)	36 (37.1)	< 0.001

IABP, intra-aotic balloon pump; CABG, coronary artery bypass grafting; EF, left ventricular ejection fraction

**Table 4:** Follow-up results

Variable	Non-CTO (n = 226)	CTO (n = 97)	OR	95% CI	*P* value
Hospital stay, days	10.1 ± 4.9	13.8 ± 7.5	-	–	< 0.001
*Events at 30 days*					
All-cause mortality mortality(n,%)	1 (0.4)	6 (6.2)	4.98	0.81–30.64	0.001
Stroke (n, %)	4 (1.8)	2 (2.1)	1.05	0.59–1.86	0.859
Re-infarction (n, %)	1 (0.4)	0	0.69	0.65–0.75	0.512
MACCE (n, %)	6 (2.7)	8 (8.3)	1.66	0.90–3.01	0.024
*Events at 10 years*					
All-cause mortality(n,%)	9 (3.9)	17 (17.5)	2.02	1.19–3.43	< 0.001
Re-infarction (n, %)	12 (5.3)	17 (17.5)	1.76	1.13–2.73	< 0.001
Stroke (n, %)	9 (3.9)	7 (7.2)	1.26	0.81–1.95	0.218
Revascularization (n,%)	52 (23.0)	17 (17.5)	0.91	0.78–1.07	0.272
PCI (n, %)	50 (22.1)	10 (10.3)	0.80	0.69–0.93	0.012
CABG (n, %)	2 (0.9)	7 (7.2)	3.21	0.94–10.92	0.002
MACCE (n, %)	77 (34.1)	51 (52.6)	1.28	1.09–1.51	0.002

PCI, percutaneous coronary intervention; CABG, coronary artery bypass grafting; MACCE, major adverse cardiocelebral events

### Follow-up results

The average follow-up time for all subjects was 123.3 ± 18.6 months. Two cases were lost in the CTO group, and four cases were lost in the non-CTO group. The follow-up rate was 98.1%. There were no significant differences between the two groups in terms of incidence of stroke or recurrent myocardial infarction at the 30-day follow-up (*P* > 0.05). The average hospital stay of the CTO group was significantly longer than that of the non-CTO group.

During the hospitalization and ten-year follow-up period, a total of 17 patients in the CTO group were revascularized due to evidence of myocardial ischemia, which was difficult to control with medication. Among them, revascularization was successfully achieved in 10 patients with CABG and 4 patients underwent PCI. There were 3 patients failed in revascularization in the PCI intervention group. At the 10-year follow-up, the rates of all-cause mortality, reinfarction, and MACCE were significantly higher in the CTO group than they were in the non-CTO group (*P* < 0.05). There was no significant difference in the rates of stroke or revascularization in the two groups (*P* > 0.05). According to the subgroup analysis, the rate of CABG was significantly higher in the CTO group than it was in the non-CTO group (7.2% *vs* 2%, *P* = 0.002), while the rate of revascularization by PCI was significantly lower in the CTO group than it was in the non-CTO group (10.3% *vs* 22.1%,* P* = 0.01) (Table 4 and Fig. 1).

### COX regression analysis of factors impacting the long-term mortality of patients with STEMI and MVD

COX regression analysis was used to analyze the long-term cause of mortality of patients with STEMI and MVD. Univariate regression analysis showed that an age ≥ 65 years, a history of OMI, a CTO in a non-IRA, a Killip class ≥ 3, and the presence of renal insufficiency, SUB, or three-vessel disease may be related to the long-term mortality of patients with STEMI and MVD (*P* < 0.1). COX regression analysis showed that a age ≥ 65 years, a CTO in a non-IRA, a Killip class ≥ 3, and the presence of renal insufficiency and SUB were significantly correlated with 10-year mortality (*P* < 0.05) (Table 5).

**Table 5:** Predictors of 10-year mortality for STEMI with MVD (n = 323) by cox regression

Variable	Univariate regression	*P* value	COX regression	*P* value
	OR (95% CI)		OR(95%CI)	
Age ≥ 65 years	3.40 (1.48–7.83)	< 0.01	3.94 (1.47–10.56)	0.01
Female	1.03 (0.41–2.57)	0.95	2.21 (0.72–6.79)	0.17
Hypertension	0.85 (0.39–1.83)	0.67	1.19 (0.48–2.95)	0.70
Diabetes	0.87 (0.36–2.06)	0.75	0.86 (0.32–2.30)	0.76
Smoking history	0.59 (0.23–2.56)	0.49	0.95 (0.57–2.01)	0.81
Hyperlipidemia	1.02 (0.41–2.55)	0.96	1.62 (0.56–4.68)	0.38
OMI	0.25 (1.21–8.73)	0.02	0.66 (0.18–2.47)	0.54
With CTO	8.71 (3.68–20.5.9)	< 0.001	5.09 (1.79–14.54)	< 0.01
Killip class ≥ 3	3.65 (1.69–7.88)	< 0.01	4.32 (1.71–10.95)	< 0.01
Renal insufficiency	7.02 (2.38–20.69)	< 0.01	5.32 (1.49–19.01)	0.01
Lung infection	3.78 (0.58–9.02)	0.09	1.82 (0.67–4.93)	0.24
Abnormal liver function	0.43 (0.06–3.13)	0.40	0.39 (0.04–3.81)	0.42
Stress ulcer	5.11 (1.53–17.12)	< 0.008	6.36 (1.45–28.01)	0.01
Three vessel disease	2.86 (0.98–8.34)	0.05	1.57 (0.46–5.26)	0.47
LAD culprit	0.60 (0.25–1.44)	0.25	0.75 (0.29–1.87)	0.53

MVD, multivessel diseases; OMI, old myocardial infarction; CTO, chronic total occlusion; LAD, left anterior descending artery

### COX regression analysis of factors impacting long-term mortality of patients with STEMI with a CTO in a non-IRA

COX regression analysis was used to analyze the long-term cause of mortality of patients with STEMI with a CTO in a non-IRA. Univariate regression analysis showed that an age ≥ 65 years, a Killip class ≥ 3, and the presence of renal insufficiency or SUB may be related to 10-year mortality (*P* < 0.1). COX regression analysis showed that Killip class ≥ 3 and the presence of renal insufficiency were significantly correlated with 10-year mortality (*P* < 0.05) (Table 6).

**Table 6:** Predictors of 10-year mortality for STEMI with a CTO in non- IRA(n = 97) by cox regression

Variable	Univariate regression	*P* value	COX regression	*P* value
	OR (95% CI)		OR (95% CI)	
Age ≥ 65 years	3.25 (1.05–10.08)	0.04	2.78 (0.69–11.24)	0.15
Female	0.72 (0.23–2.2)	0.56	1.67 (0.37–7.57)	0.51
Hypertension	0.61 (0.23–1.60)	0.31	0.96 (0.29–3.18)	0.95
Diabetes	0.79 (0.28–2.27)	0.67	1.25 (0.32–4.83)	0.75
Smoking history	0.91 (0.33–2.78)	0.57	0.83 (0.46–3.86)	0.68
Hyperlipidemia	0.52 (0.18–1.7)	0.22	0.70 (0.16–3.09)	0.64
Killip class ≥ 3	4.34 (1.59–11.78)	< 0.01	2.97 (1.46–6.03)	< 0.01
Renal insufficiency	3.93 (1.28–12.06)	0.01	5.61 (1.19–26.39)	0.03
Lung infection	0.84 (0.27–2.58)	0.76	0.61 (0.15–2.56)	0.49
Abnormal liver function	1.14 (0.15–8.71)	0.89	0.76 (0.04–13.67)	0.86
Stress ulcer	8.25 (2.31–29.43)	< 0.01	3.33 (0.47–24.37)	0.24
Three vessel disease	1.24 (0.28–5.44)	0.78	0.74 (0.12–4.57)	0.75
LAD culprit	0.74 (0.24–2.29)	0.59	0.85 (0.22–3.42)	0.83

OMI, old myocardial infarction; CTO, chronic total occlusion; LAD, left anterior descending artery; IRA, infarct-related artery

## Discussion

The purpose of this study was to examine the long-term outcome of patients with STEMI and MVD that were treated with primary PCI, comparing the outcomes of patients with and without a CTO in a non-IRA and evaluating the impact of the presence of a CTO on the patient’s long-term prognosis. After an average follow-up period of 123.3 months, the primary findings of this study were as follows. First, the presence of a CTO in a non-IRA, an age ≥ 65 years, severe heart failure during hospitalization, and the presence of renal insufficiency increased the long-term mortality of patients with STEMI and MVD; these factors were determined to be independent predictors of long-term mortality. After adjusting for differences in the patients’ baseline clinical characteristics, the presence of a CTO in a non-IRA remained a strong independent predictor of increased mortality in patients with STEMI. Second, patients in the CTO group that experienced severe heart failure and renal insufficiency during hospitalization had a worse long-term prognosis than the other patients in the CTO group.

During hospitalization, the rates of mortality and MACCE were higher in patients with STEMI that had a CTO in a non-IRA than they were in patients without a CTO. All of the patients enrolled in the study were treated in accordance with the guidelines that were current at the time of treatment. In patients without a hemodynamic disorder, intervention was only performed on the culprit vessels during emergent PCI, and patients that did not have the symptoms of myocardial ischemia during hospitalization did not undergo revascularization [10,11]. The treatment strategy for patients that required further revascularization was jointly formulated by cardiologists and surgeons [10–12] during hospitalization. During the hospitalization, 4 patients in the non-CTO group received revascularization: 1 suffered from sub-acute thrombus and underwent emergent PCI once again, and 3 received CABG. By contrast, 10 patients in the CTO group received CABG since the SYNTAX scores of these patients are all above 35. Due to anatomical factors such as calcification, tortuosity, and bifurcation, PCI was risky and CABG was more suitable for these patients. The rate of symptom-driven revascularization was significantly higher in the CTO group than it was in the non-CTO group, which indicates that patients with STEMI and a CTO in a non-IRA are more likely to suffer from AMI and require revascularization. All of the patients that received revascularization for a second time survived the hospitalization period. At the 30-day follow-up after the primary PCI, 7 patients had died, where 6 of these deaths occurred in the CTO group. The rates of mortality and MACCE were higher in the CTO group than they were in the non-CTO group. During hospitalization, STEMI patients with a CTO in an IRA had a slightly higher incidence of stress ulcers than was experienced by the patients without a CTO, but this difference was not significant. These results show that the occurrence of stress ulcers may be related to the severity of cardiac dysfunction. Because the incidence of stress ulcers was elatively high (3.4%), no significant correlation was found in our study.

At the 10-year follow-up, the mortality and MACCE rates were significantly higher in the CTO group than they were in the non-CTO group: The risk of all-cause death was 2.02 times higher in patients with a CTO, and the risk of MACCE was 1.66 times higher. In addition, the risk of recurrent myocardial infarction was 1.76 times higher in patients with a CTO than it was in patients without a CTO, which suggests that there was a high long-term risk of re-infarction. There was no significant difference in the two groups in terms of stroke risk. Although the overall revascularization rate was similar in the two groups, the rate of revascularization by PCI was significantly higher in the non-CTO group, and the rate of CABG was significantly higher in the CTO group. This indicate that a greater percentage of patients with a CTO opted for treatment with CABG, whereas a greater percentage of patients without a CTO opted for treatment with PCI. The management of STEMI patients with CTO in a non-IRA still have some debate concerning the necessity of PCI and optimal timing of PCI (immediate *vs.* staged). The recent COMPLETE trial demonstrated that a clinical benefit of staged PCI of non-culprit lesion was better than the strategy of culprit-lesion-only PCI [13], and using FFR (fractional flow reserve), iFR (instantaneous wave-free ratio) and non-invasive imaging techniques may be essential for the assessment of residual ischaemia deriving from non-IRA and the optimal timing for PCI [14].The results of this study are consistent with those of the Harmonizing Outcomes with Revascularization and Stents in AMI (HORIZONS-AMI) trial discussed by Claessen et al. [15]. The trial found that the 30-day and 3-year mortality rates were significantly higher for patients with STEMI and a CTO than they were for patients without a CTO that had either MVD or single-vessel disease [15]. The Intra-aortic Balloon Pump in Cardiogenic Shock II (IABP-SHOCK II) trial published by Saad et al. [16] reported that the presence of a CTO in a non-IRA was an independent predictor of the 12-month mortality of patients with cardiogenic shock and AMI. The results of the 10-year follow-up period used in our study demonstrate that the differences in the rates of mortality and MACCE persisted in the long term, where patients with a CTO in a non-IRA had worse outcomes.

Our study showed that patients in the CTO and non-CTO groups did not differ in terms of age, sex composition ratio, the rate of previous PCI, or the risk factors of hypertension, diabetes, hyperlipidemia, and smoking. Patients with STEMI and a CTO in a non-IRA had higher incidences of OMI, renal insufficiency, cardiac insufficiency, and lung infection. Patients with a CTO are more likely to suffer from the decompensation of cardiac function because a greater amount of viable myocardium must be available for the culprit vessels to provide collateral supply during an AMI. When an occlusion occurs in a culprit vessel, it induces multiple infarction and leads to greater impairment of cardiac function. The Killip classification of cardiac function reflects the severity of cardiac dysfunction in patients with AMI; these classifications are consistent with the clinical manifestations of AMI and the results of chest X-rays. Because the assignment of Killip classes by different doctors produces reliable results with greater consistency and less variance, Killip classification was used in this study to analyze the cardiac function of patients during the acute phase of STEMI. It was found that patients with STEMI and a CTO were characterized as Killip class 3 or 4 at a significantly higher rate than patients without a CTO were. The occurrence of severe cardiac dysfunction was an independent predictor of long-term mortality in all patients with STEMI and MVD, both those with a CTO and those without a CTO.

### Limitations

This study had several limitations. First, it was a retrospective study and did not consider whether the successful intervention of a CTO in a non-IRA would improve the outcome of patients with STEMI. In a retrospective study by Deng et al. [17], 377 STEMI patients with CTO lesions in a non-IRA underwent early PCI intervention. The results showed that the rates of all-cause mortality and MACCE after one year were significantly lower in patients for whom intervention was successful than they were in patients that did not receive intervention or who failed in operation. However, the results of the EXPLORE trial showed that the left ventricular function of patients with STEMI and a CTO did not improve even when PCI intervention was conducted as soon as one week after the primary procedure; in addition, the all-cause mortality rate did not decrease after five years of follow-up, and the cardiogenic mortality rate was even higher in these patients than it was in the patients in the medication treatment group. In addition, patients in whom CTO lesions were successfully opened only experienced a lower rate of angina for one year after the operation, and there was no difference between the rates of angina in the two groups in the subsequent four years [18]. Future work such as appropriate controlled randomized studies with detailed information can be adopted to further clarify the benefit of staged PCI for the STEMI patients with a CTO in a non-IRA.

The inconsistent results of the previous clinical studies reflect the difficulty of treating patients with STEMI and a CTO in a non-IRA. The group of patients enrolled in the present study had more factors that could adversely affect their outcome, namely older age, OMI with chronic heart failure, and the presence of renal insufficiency. As a result, revascularization via PCI or CABG was more likely to be poorly tolerated and have a higher rate of complications or failure. There have been no clinical trials designed to determine whether a CTO in a non-IRA is best treated when PCI is initially performed or a short time later. Given the complexity and difficulty of treating patients with a CTO and the adverse effects of cardiac function impairment, the choice between PCI and CABG should be made on the basis of the patient’s specific clinical and pathological characteristics and the input of the operating surgeons. For patients with STEMI in whom the IRA is the left anterior descending branch, the results of the EXPLORE trial suggest that the IRA should be opened as quickly as possible, since doing so can benefit the patient. Second, this study did not include patients that underwent complete revascularization without objective evidence of ischemia in the same hospitalization period. Previous clinical trials (Preventive Angioplasty in Acute Myocardial Infarction [PRAMI], Complete versus Lesion-only Primary PCI Trial [CvLPRIT], and the Third Danish Study of Optimal Acute Treatment of Patients with STEMI and Primary PCI in Multivessel Disease [DANAMI-3–PRIMULTI]) [19–21] observed that when patients with STEMI and MVD received treatment for non-IRAs in the setting of emergent PCI, the rate of long-term revascularization was lower than it was when only IRAs were treated. However, there were no changes in the rates of all-cause mortality or non-fatal myocardial infarction during the follow-up. On the basis of the results of these trials, the guidelines of the European Society of Cardiology and the American College of Cardiology have increased their level of recommendation for the treatment of non-IRAs during emergent PCI; however, this treatment still does not receive a class I recommendation from either organization [22,23]. Because our study screened patients that had been treated before the results of these trials were released, we limited our attention to the long-term outcomes of patients that were treated according to the guidelines current at the time of treatment; as a result, our study excluded patients that underwent complete revascularization on the basis of visual judgment alone, with no objective evidence of myocardial ischemia in the same hospitalization period.

### Conclusions

The results of our study demonstrated that patients with STEMI and a CTO in a non-IRA have worse outcomes than patients without a CTO. In patients with STEMI and MVD, the presence of a CTO in a non-IRA was found to be a predictor of both short- and long-term mortality. In patients with a CTO in a non-IRA, an in-hospital Killip class ≥ 3 and the presence of renal insufficiency were found to be independent risk predictors of both short- and long-term mortality.

Both short- and long-term treatment strategies should focus on reducing the rates of mortality and angina and preventing recurrent myocardial infarction. Before revascularization takes place, the viable myocardium in the supply area of the vessel in which the CTO has developed should be evaluated in terms of the sites of collateral circulation (their number, source, classification, and location and whether they involve ipsilateral or reverse collaterals) and the degree of intraoperative risk (including patient tolerance and the complexity of the lesions). If a case is determined to be suitable for revascularization with PCI or CABG, then the vessel containing the CTO and any other affected vessels should be rebuilt. If a case is determined to be unsuitable for revascularization, then lifestyle intervention and optimal medical therapy should be recommended for the duration of the patient’s life.

## Data Availability

We declared that materials described in the manuscript, including all relevant raw data, will be freely available to any scientist wishing to use them for non-commercial purposes, without breaching participant confidentiality.

## Abbreviations

AMI: Acute myocardial infarction
CABG: Coronary artery bypass grafting
CTO: Chronic total occlusion
SUB: Stress ulcer with bleeding
FFR: Fractional flow reserve
iFR: Instantaneous wave-free ratio
IRA: Infarct-related artery
MACCE: Major adverse cardiovascular and cerebrovascular events
MVD: Multivessel diseases
OMI: Old myocardial infarction
PCI: Percutaneous coronary intervention
STEMI: ST-segment elevation myocardial infarction
TIMI: Thrombolysis in Myocardial Infarction
